# Collusion-Resistant Audio Fingerprinting System in the Modulated Complex Lapped Transform Domain

**DOI:** 10.1371/journal.pone.0065985

**Published:** 2013-06-06

**Authors:** Jose Juan Garcia-Hernandez, Claudia Feregrino-Uribe, Rene Cumplido

**Affiliations:** 1 Laboratorio de Tecnologias de Informacion, CINVESTAV-IPN, Tamaulipas, Mexico; 2 Instituto Nacional de Astrofisica, Optica y Electronica, Puebla, Mexico; UC Davis School of Medicine, United States of America

## Abstract

Collusion-resistant fingerprinting paradigm seems to be a practical solution to the piracy problem as it allows media owners to detect any unauthorized copy and trace it back to the dishonest users. Despite the billionaire losses in the music industry, most of the collusion-resistant fingerprinting systems are devoted to digital images and very few to audio signals. In this paper, state-of-the-art collusion-resistant fingerprinting ideas are extended to audio signals and the corresponding parameters and operation conditions are proposed. Moreover, in order to carry out fingerprint detection using just a fraction of the pirate audio clip, block-based embedding and its corresponding detector is proposed. Extensive simulations show the robustness of the proposed system against average collusion attack. Moreover, by using an efficient Fast Fourier Transform core and standard computer machines it is shown that the proposed system is suitable for real-world scenarios.

## Introduction

In the Information Technology era, expansion of the Internet service together with the rapid advance of high capacity storage systems facilitated the fast and perfect copy of digital content. However, at the same time the use of these technologies causes serious problems, such as unauthorized copying and distribution of digital materials, [1]. Conventional cryptography systems encrypt digital data during its transmission and permit only authorized person to decrypt the encrypted data, nevertheless, once such data are decrypted they are totally vulnerable to illegal copying and distribution. One possible solution to this problem is the fingerprinting paradigm, where, a unique signature (which identifies to the legal user) known as a digital fingerprint is hidden using a watermarking technique into the content previously to distribution. Watermarking has several applications such as: ownership proof [2], secret communications [3], bio-security [4], [5], etc. Digital fingerprinting, which is also a watermarking application, has the capacity of identifying illegal users by extracting the fingerprint of a suspicious copy. A typical attack in fingerprinting systems is the collusion attack, where a group of users combine their copies in order to remove the original fingerprint. If a sufficient number of copies are combined, the noise produced by the collusion attack can disable/confuse the fingerprint detector and prevent the content owner from identifying the illegal users. Although several linear and nonlinear operations can be utilized for a collusion attack, it has been shown that the worst one is the linear averaging [6]. Therefore, it is necessary to design collusion-resistant fingerprints that can identify the greatest number of colluders involved in a pirate copy.

Collusion-resistant fingerprint codes have been proposed as a solution to the collusion attack [6]–[9]. Theoretical results for collusion-resistant fingerprint codes have shown interesting properties against collusion attacks, however, in practical sceneries their performance needs further research as these can be sensible to other kinds of attacks, [9].

On the other hand, Spread Spectrum (SS) modulation is a watermarking technique that has shown to be remarkably robust to several attacks, collusion included, [10]–[13]; therefore, it has been frequently utilized in fingerprinting systems [14]–[16]. The main drawback of fingerprinting schemes based in spread spectrum modulation is their high computational complexity as the number of correlations performed is proportional to the number of possible users. A users grouping approach was proposed in [17]. That idea is based on the consideration of colluders being more likely to have similar geographical area and interests with each other. Users are grouped according to common conditions between them. When a suspicious copy is identified, the first search is about the group IDs and then for user' IDs. The computational complexity is reduced, due to the colluders search is carried out in a tree fashion, [17].

In [18], the use of PN-modulated Discrete Cosine Transform (DCT) basis as fingerprints for digital images is proposed. The DCT operation can be represented as a multiplication between the input vector and one matrix conformed by the DCT basis. That multiplication is equivalent to correlations between the input vector and each column of the DCT matrix. Therefore, a fast DCT algorithm reduces the computational complexity of correlations needed in the IDs detection to the logarithmic scale. The fingerprint is formed by the sum of two PN-modulated DCT basis, one for the group ID and the other for the user ID. In the detection stage, firstly the groups to which colluders belong are detected, and then colluders are detected for each of them. In [19] the interference due to colluder fingerprints is removed and performance of the system in [18] is improved drastically.

The music piracy produces large monetary losses around the world [20], [21], therefore, a tool that helps to mitigate the music piracy is mandatory. However, most of the reported collusion-resistant fingerprinting schemes are devoted to digital images [7], [8], [16], [18], [19], and only very few are validated with audio signals [24]. This paper is about collusion-resistant audio fingerprinting. A collusion-resistant audio fingerprinting system based on some of the ideas developed for digital images in [18], [19] is proposed. Instead of using the full signal as [18], [19] a block-based fingerprint embedding strategy is followed and the corresponding detector is derived. In this paper, the Modulated Complex Lapped Transform (MCLT) domain is utilized as fingerprint channel due to no block-artifact property in audio watermarking systems [11], [25]–[28].

### Related Work

Work reported in [24] claims to be able to detect 80 colluders in a pirate audio clip. However, that system seems to be not suitable for real world scenarios. One weakness is about construction of component vector which is carried out using two audio channels in the Fourier domain. Due to a trigonometric function (inverse tangent) is involved in this stage, a simple attack like sign inversion in one audio channel prevents the correct ID detection as multiplication by −1 is equivalent to a phase shift by 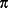 radians. Moreover, if an audio channel is scaled (volume gain) the relation between both channels will be different to the original and the detection will fail. Detector performance after lossy compression, such as MP3 coding or Advanced Audio Coding (AAC) is not reported. It is important to mention that sign inversion, volume gain and lossy compression are real world scenarios. Neither viability of the system nor the number of users is reported. To the best of our knowledge, it is the only work about collusion-attack resistant fingerprinting in audio signals. Although there are several works about audio fingerprinting in the literature, almost all of them do not consider the collusion-attack [29]–[33].

**Figure 1:**
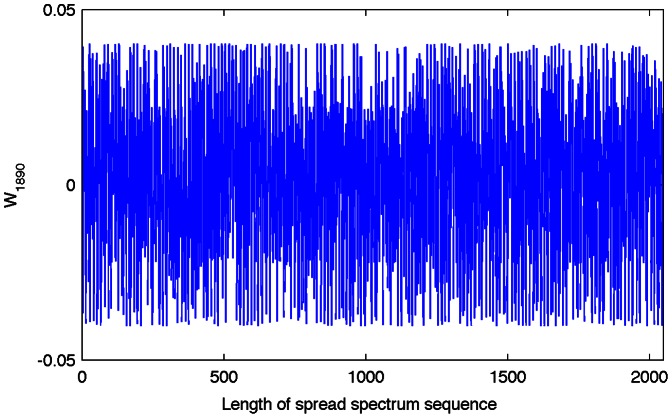
Spread spectrum sequence for the user 1890 of 2048.

On the other hand, most of the works about collusion-attack resistant fingerprinting systems are devoted to digital images mainly based in Spread Spectrum techniques. The main drawback of fingerprinting schemes based in SS techniques is their high computational complexity as it is discussed in the Introduction. In order to achieve lower computational complexity than SS-fingerprinting schemes for digital images, in [18] it is proposed to utilize PN-modulated orthogonal sequences. These orthogonal sequences can be obtained from DCT or DFT basis. In the DCT case, each user is related to a DCT matrix column which is defined in equation (26). Therefore, the SS sequence for the 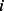
th user becomes:
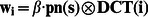
(1)


where 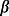 is a robustness factor, 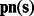 is a PN sequence generated using an initial value 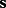, 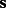 is a secret key, 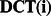 is the 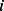
th DCT matrix column and 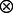 is the element-wise multiplication. The sequence 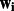 is embedded into the frequency components of a digital medium, in this paper audio signals. As an example, Figure 1 shows the SS sequence, 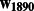, for the user 1890 of 2048 and 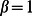.

**Figure 2:**
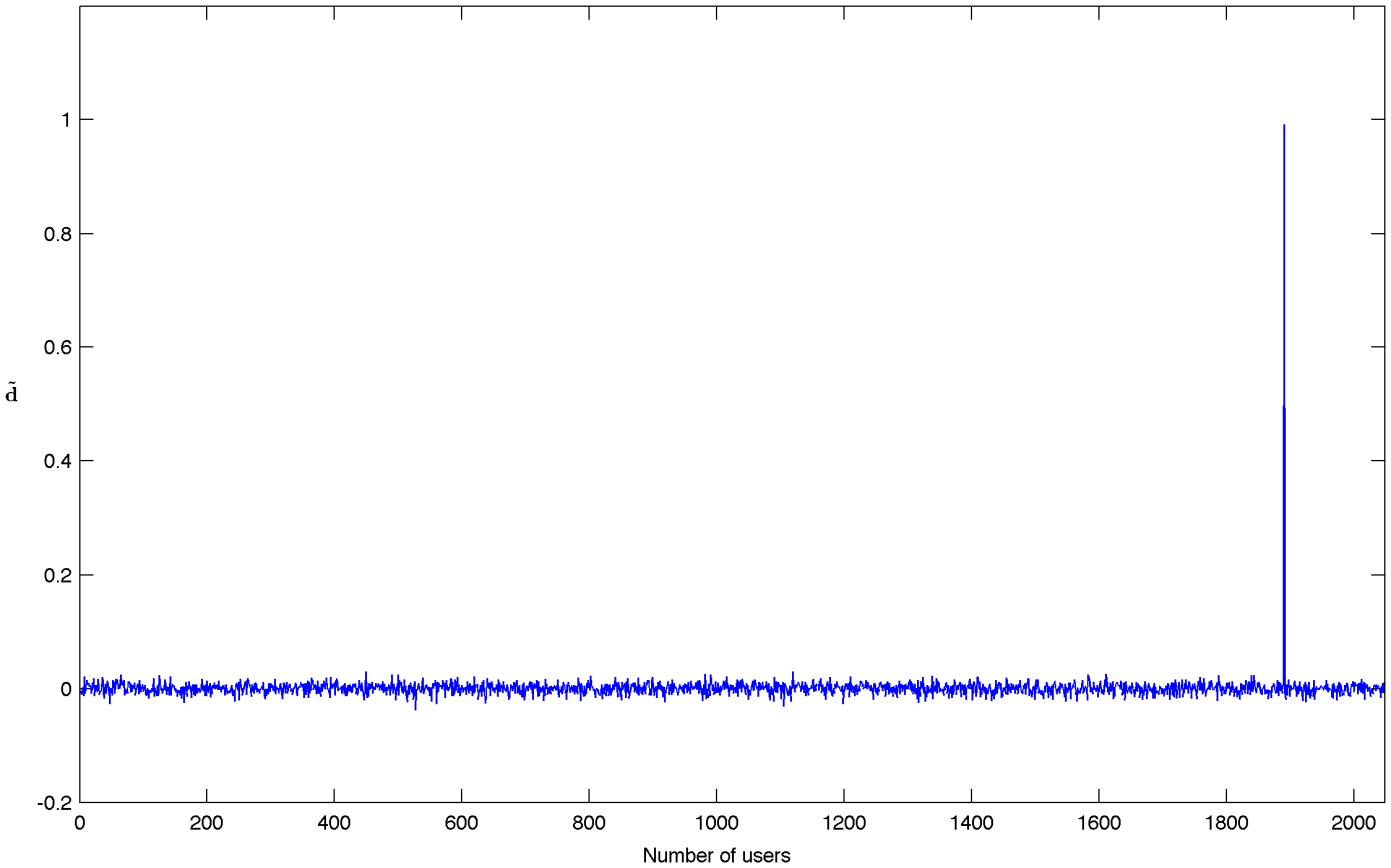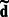 for detection of the user 1890 of 2048.

Unlike other watermarking applications, in the fingerprinting paradigm, detection is usually carried out in a non-blind fashion [34], i.e. the original signal is available to the detector. Under that condition, after subtracting the original sequence from the pirate copy the sequence 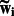 is obtained. In order to carry out the detection the sequence 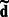 is obtained by applying the Inverse DCT to 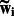 which is demodulated by the PN sequence 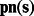 as follows:
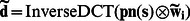
(2)where InverseDCT(.) denotes a fast inverse discrete cosine transform algorithm as described in the Materials and Methods section. Figure 2 shows the corresponding 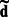 for detection of the user 1890 out of 2048, as exemplified above.

From Figure 2, it is possible to observe that a threshold is necessary in order to determine the user under a statistical point of view. If 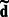 is supposed to be 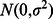 except for a fingerprinted component 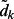, it is possible to calculate a threshold 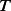 according to the probability of false detection 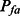 [18] as follows:
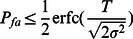
(3)


where erfc() is the complementary error function defined as:
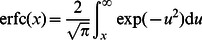
(4)


Therefore, the threshold is given by the expression (5),
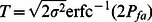
(5)where erfc
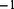
(.) stands for the inverse complementary error function.

Grouping a set of users has been proposed in the literature as a solution to high computational costs [8], [16]. The assumption behind this proposal is that users who have a similar background and region are more likely to collude each other. In [18] the idea of introducing dependency between two SS sequences by exploiting the property of quasi-orthogonality of PN sequences is proposed. Thus, the fingerprint is integrated by two spread spectrum sequences related to a group ID 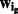 and an user ID 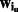 as follows:
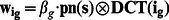
(6)


where 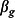 is the robustness factor for groups, 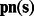 is a PN sequence generated with the secret key 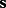, 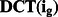 is the 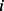
th basis vector that identified to the 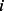
th group and
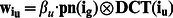
(7)


where 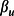 is the robustness factor for users, 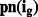 is a PN sequence corresponding to the 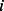
th group, and 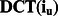 is the 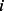
th basis vector that identified the 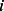
th user.

Then, the fingerprint assigned to the 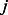
th user of the 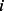
th group is conformed by:
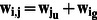
(8)


The energy of the fingerprint is represented by
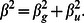
(9)


From equation (8) it is easy to see that a couple of detectors is required, one for the spread spectrum sequence related to group ID 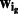 and other for the user ID 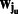. These detectors are derived from equation (2) as follows:

For group ID detection:
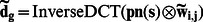
(10)


and for user ID detection:
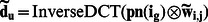
(11)


with thresholds, 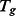 and 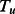, derived according to equation (5) as follows:
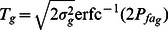
(12)

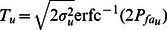
(13)


where 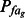 and 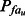 are given false positive probabilities for the group and user ID detection procedures respectively. 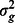 and 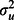 are the variance of the group and user ID detection sequences respectively.

The outline of the paper is as follows: First, experimental results and discussion are offered. In the Materials and Methods section, we recall the Modulated Complex Lapped Transform and Discrete Cosine Transform and their fast algorithms used in this work. In the Fingerprinting System section steps are described comprising audio fingerprinting method by DCT modulation in the MCLT domain. Finally, conclusions are offered.

## Results and Discussion

The proposed audio fingerprinting system is evaluated under averaging collusion attacks. Through abundant experiments; the operation parameters are determinate too. For experimentations, CD-quality audio files are utilized from a set of 1000 popular music recordings. The probability of false detection is set to 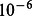 for both group (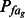) and user ID detection (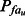) procedures, as this is a typical value in audio spread spectrum-based watermarking systems [11].

### Fingerprint Robustness Determination

**Figure 3:**
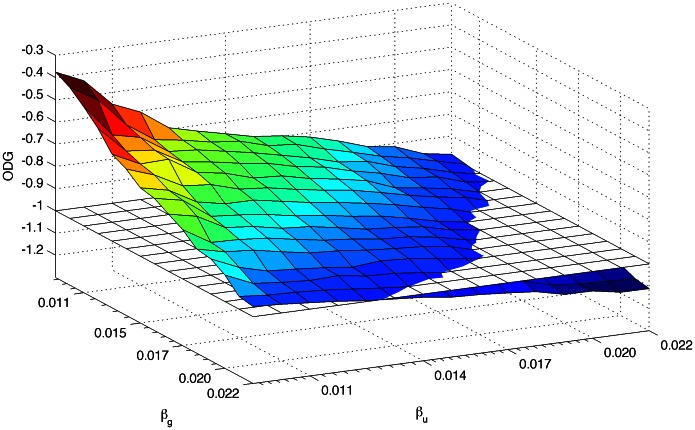
ODG region for fingerprinted audio clips.

In order to determinate the adequate 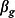 and 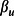 values in equations (6) and (7); an audio transparency metric is utilized, the Objective Difference Grade (ODG) [35]. An ODG value between 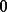 and −1 is considered a good perceptual transparency [35]. In the experiment, several audio clips are fingerprinted with different combinations of 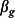 and 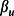 values and the ODG metric for each combination is obtained. The limit for practical 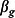 and 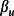 values is determinate for ODG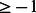 as the bigger the fingerprint energy 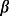 the lower the ODG value. In the spread spectrum watermarking, it is well known that the bigger the watermark energy the bigger the watermark robustness [2]. Therefore, it is interesting to investigate the biggest fingerprint energy values that maintain a good perceptual transparency. Figure 3 shows the ODG region for an average of 10 sets of 225 fingerprinted audio clips.

In order to provide a reference for practical 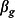 and 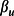 values, Table 1 shows the corresponding ODG values for combinations of 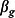 and 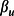 values.

**Table 1:** ODG values for combinations of 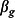 and 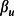 values.

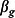 / 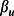	0.0008	0.0009	0.0010	0.0011	0.0012	0.0013	0.0014	0.0015	0.0016	0.0017	0.0018	0.0019	0.0020	0.0021	0.0022
0.0008	–0.38	–0.41	–0.47	–0.53	–0.61	–0.64	–0.69	–0.71	–0.74	–0.78	–0.83	–0.84	–0.88	–0.94	–0.95
0.0009	–0.43	–0.49	–0.61	–0.65	–0.67	–0.69	–0.72	–0.73	–0.77	–0.85	–0.86	–0.90	–0.94	–0.95	–0.96
0.0010	–0.55	–0.61	–0.64	–0.66	–0.67	–0.75	–0.79	–0.82	–0.84	–0.86	–0.87	–0.93	–0.95	–0.96	–0.97
0.0011	–0.61	–0.70	–0.72	–0.73	–0.74	–0.76	–0.82	–0.83	–0.85	–0.87	–0.89	–0.94	–0.96	–0.97	–0.98
0.0012	–0.69	–0.72	–0.73	–0.75	–0.76	–0.80	–0.82	–0.85	–0.87	–0.89	–0.92	–0.95	–0.95	–0.98	–1.00
0.0013	–0.73	–0.74	–0.75	–0.77	–0.80	–0.81	–0.84	–0.87	–0.88	–0.91	–0.92	–0.96	–0.97	–0.99	–1.01
0.0014	–0.76	–0.75	–0.77	–0.79	–0.81	–0.84	–0.86	–0.88	–0.91	–0.92	–0.94	–0.95	–0.97	–1.00	–1.01
0.0015	–0.78	–0.79	–0.80	–0.82	–0.84	–0.86	–0.88	–0.91	–0.93	–0.94	–0.95	–0.96	–0.99	–1.02	–1.03
0.0016	–0.80	–0.80	–0.83	–0.84	–0.86	–0.87	–0.90	–0.92	–0.94	–0.95	–0.96	–0.98	–1.01	–1.01	–1.04
0.0017	–0.82	–0.84	–0.87	–0.87	–0.88	–0.90	–0.92	–0.95	–0.95	–0.97	–0.97	–0.99	–1.02	–1.03	–1.05
0.0018	–0.86	–0.87	–0.88	–0.90	–0.93	–0.93	–0.95	–0.98	–0.98	–0.99	–1.01	–1.01	–1.04	–1.04	–1.06
0.0019	–0.90	–0.91	–0.92	–0.94	–0.95	–0.95	–0.97	–1.00	–1.00	–1.01	–1.02	–1.02	–1.04	–1.08	–1.09
0.0020	–0.92	–0.93	–0.94	–0.95	–0.97	–0.97	–1.01	–1.02	–1.04	–1.05	–1.06	–1.05	–1.06	–1.09	–1.09
0.0021	–0.96	–0.96	–0.98	–0.99	–1.00	–1.01	–1.02	–1.03	–1.05	–1.06	–1.07	–1.06	–1.07	–1.10	–1.11
0.0022	–0.98	–0.99	–1.00	–1.00	–1.02	–1.03	–1.05	–1.05	–1.07	–1.06	–1.07	–1.06	–1.07	–1.10	–1.11

**Figure 4:**
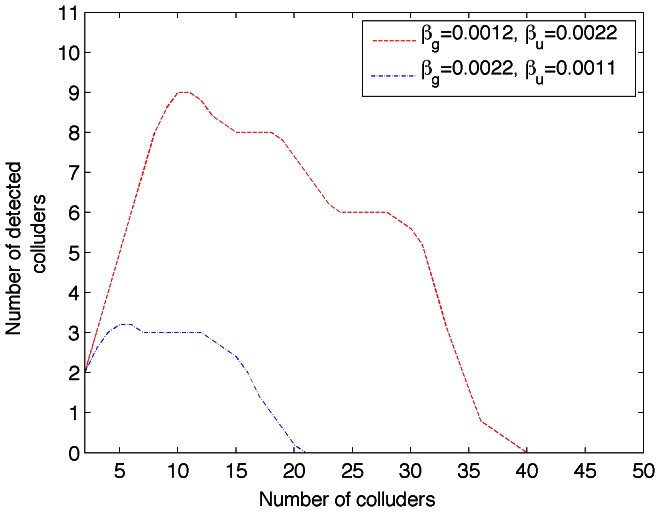
Collusion-attack robustness for 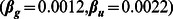 and 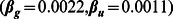.

Figure 4 shows the collusion-attack robustness for the combination with the higher acceptable 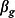 and the combination with the higher acceptable 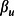, with colluders from the same group and block length, 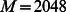. It is interesting to note that the detection performance is better when the robustness factor for users is greater than the robustness factor for groups, i.e. 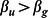, moreover, according to [17], users in a group are more likely to collude with each other, therefore, the number of group IDs involved in a pirate copy must be smaller than the number of colluder IDs. As a consequence, the energy of the user ID PN-sequence must be higher than the group ID PN-sequence, i.e. 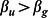. Under that asseveration, combinations 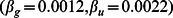, 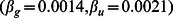, 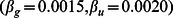 and 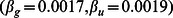 seem to be good candidates for fingerprint embedding.

### Block Length Influence

**Figure 5:**
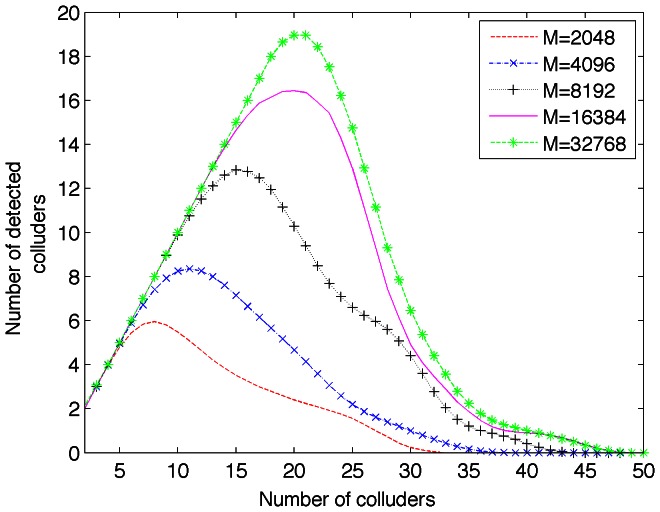
Collusion-attack robustness for several block length, 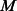.

From Figure 4 it is possible to observe that in the best case, 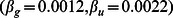, the number of detected colluders appears low for practical applications. In order to improve the performance of the proposed system, the influence of the block length, 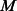, is investigated. Collusion-attack robustness is studied for different block lengths and Figure 5 shows the results for such study.

**Figure 6:**
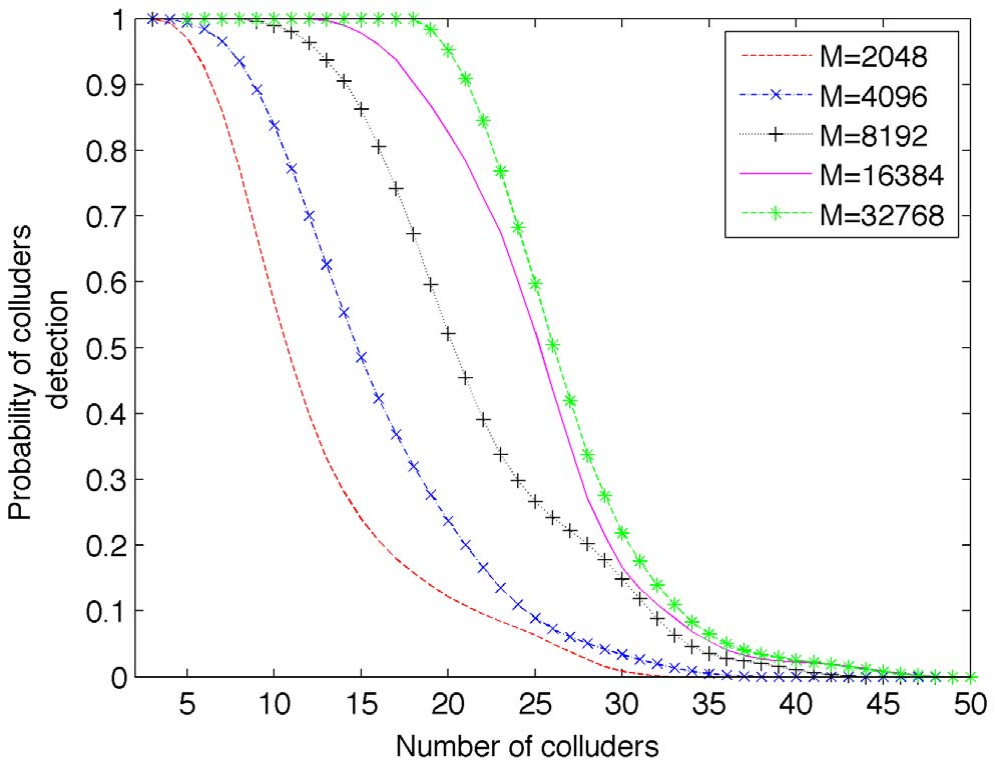
Detection rate of colluders in function of block length, 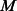.

The higher block length the higher collusion-attack robustness. However, for 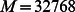 the increase in performance is not significant in comparison with 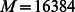 as it can be seen in Figure 5. Moreover, the computing resources for computing FFT in the MCLT and DCT transforms can be critical for some platforms when the number of points is larger [36]. Therefore, 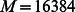, seems to be the best option as it is possible to detect more colluders users from the totality of them. Figure 6 shows the detection rate of colluders in function of block length, 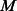. This confirms what mentioned from results in Figure 5.

### Implementation Issues

In this subsection, the viability of the proposed system is addressed. It can be interesting to measure the computing time of fingerprint embedding and detection as a function of the block sizes as several block sizes have been studied. Table 2 shows time requirements for several block sizes in real-time terms. It is interesting to point out that the computing complexity increases very slightly when the block size increases in two-power factor.

**Table 2:** Time requirements for several block sizes.

Block Size	Fingerprint embedding (real-time)	Fingerprint detection (real-time)
2048	38.04x	33.24x
4096	36.23x	32.50x
8192	35.65x	32.39x
16384	33.68x	31.36x

According to Table 2, an 80 min. music album can be fingerprinted in about 2.37 min. (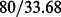) which could be attractive for on-line music distribution services.

Let 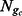 be the number of groups of colluders involved in a pirate copy, it is necessary 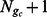 detection process operations in order to find all of the colluders as one detection process is utilized for group IDs detection and 1 detection process for each detected group in order to identify colluder user IDs. As an example, if an 80 min. music album is pirated by 40 users from 5 groups, the colluders detection in the whole album requires about 15.3 min. (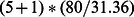) which seems to be a non-prohibitive amount of time for commercial applications. Moreover, if the number of colluders is higher but the number of groups is the same, the computational complexity will be maintained about 15.3 min. as it only depends of number of detected groups.

### Audio Clip Requirements for IDs Detection

**Figure 7:**
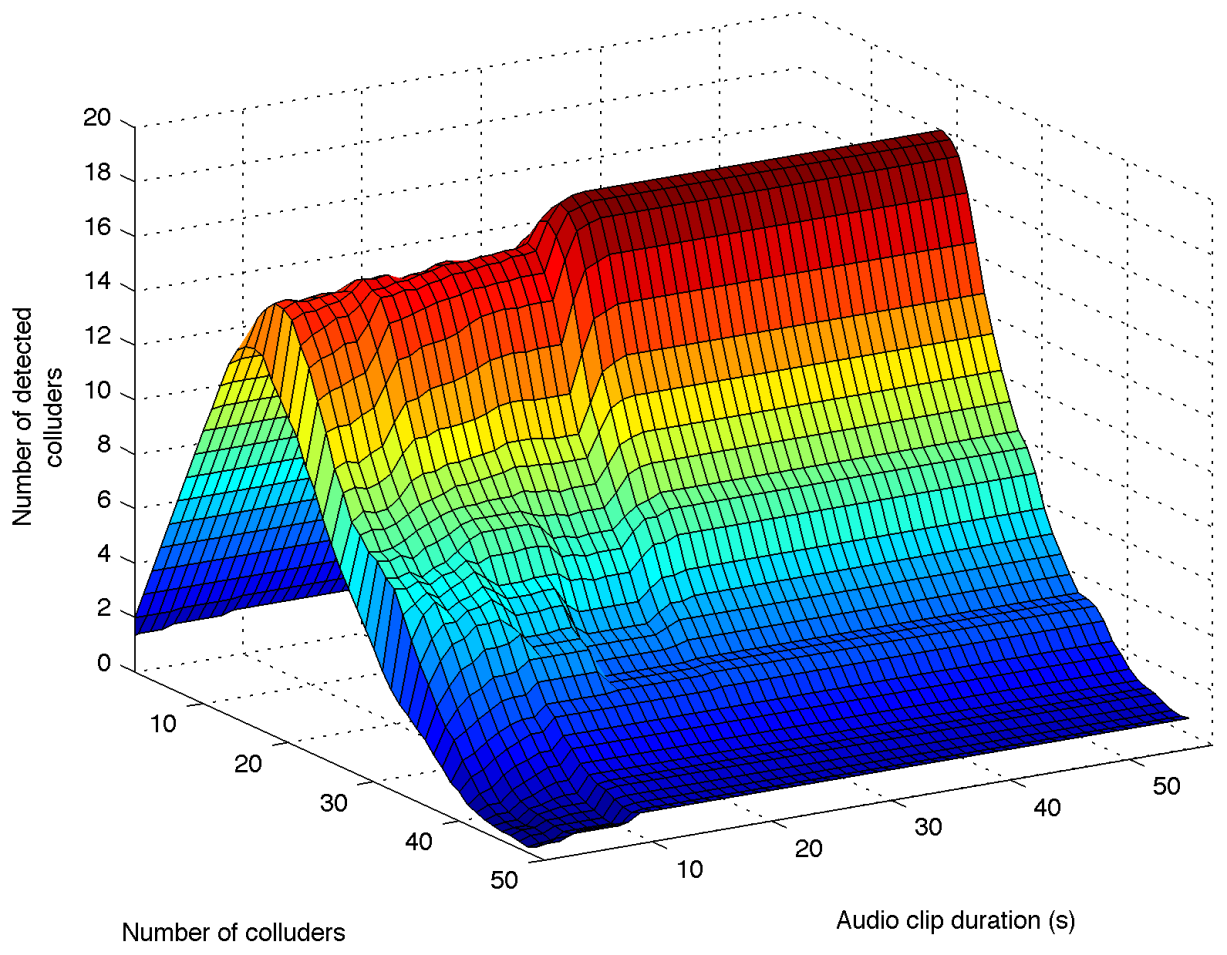
Colluders detection in function of pirate audio clip duration.

Due to the nature of the fingerprint insertion process, it is possible to assume that it is not necessary the whole audio clip in the detection process. The IDs detection is carried out by a counter of events that exceed thresholds, therefore, if there are enough events the system achieves its maximum detection capacity. This is expected to happen after a certain number of events and after the behavior of the detector goes stable. In order to validate that claim, the next experiment was carried out: a set of audio clips were fingerprinted with different IDs, and a pirate copy was generated for 2 to 50 colluders; for 1 to 55 seconds of the audio clip ID detection is executed and detected colluders are counted. This experimentation was carried out with 100 different pirate audio clips with 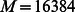 and their results are averaged. Figure 7 shows the detector behavior in function of pirate audio clip duration and number of colluders.

**Figure 8:**
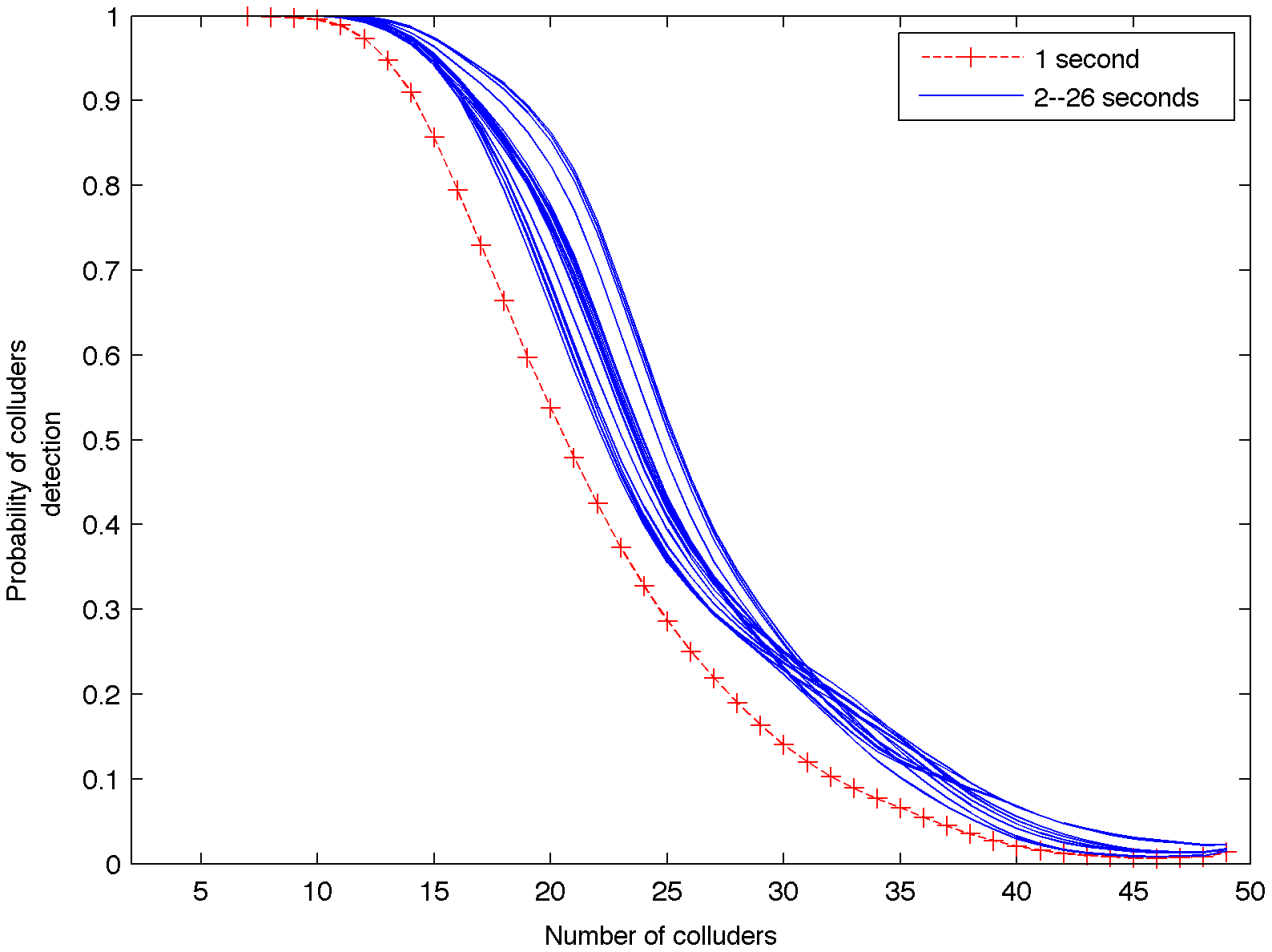
Probability of colluders detection for several pirate audio clip durations.

It is interesting to observe that the curve remains without notable changes from 26 seconds to 55 seconds. In other experimentation, using several 30 seconds pirate audio clips, the detector capacity is the same as compared with detection using the whole pirate audio clips, which corroborates the behavior shown in Figure 7. On the other hand, according to Figure 8, for a probability of colluders detection equal to 1, the detector behavior is practically the same for durations longer that 2 seconds.

### Lossy Compression Attack

**Figure 9:**
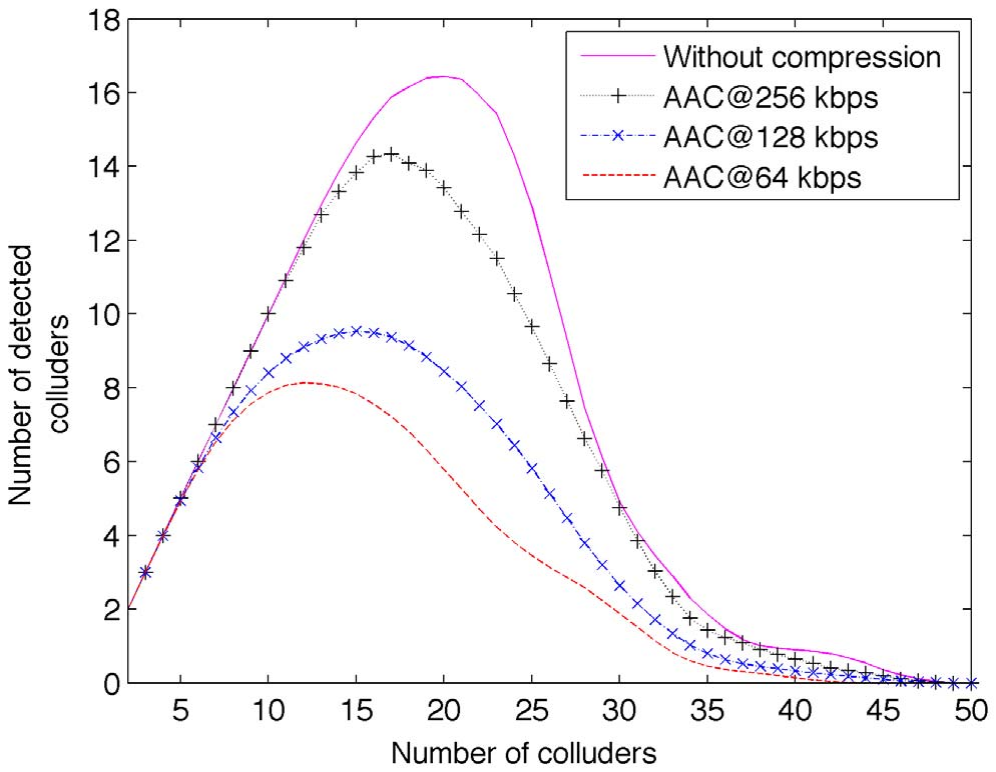
Collusion-attack robustness for several AAC bitrates.

In order to validate the proposed system in a practical scenario, robustness to collusion attack after lossy compression is explored. Advanced Audio Coding (AAC) is used for experimentation as it has shown better performance in perceptual transparency and compression rates terms as compared with MPEG-1 and MPEG-2 Audio Layer 3 [37]. The block length utilized in the experiment is 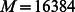, and the number of audio clips involved is 225. Figure 9 shows the detector performance under collusion attack after AAC compression for several bitrates.

**Figure 10:**
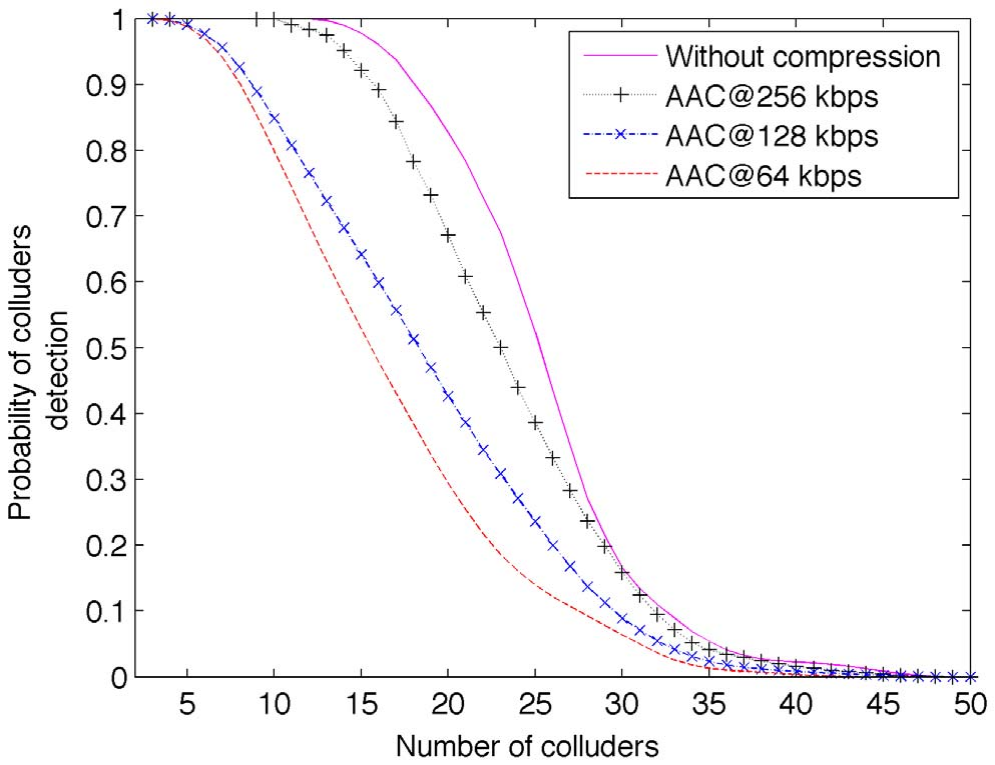
Detection rate of colluders in function of several AAC bitrates.

It is possible to see from Figure 9 that the lower AAC bitrate the lower performance. Figure 10 shows the detection probability of colluded attacked audio clip after AAC compression for several bitrates. The detector performance reduces about 12% after high quality lossy compression; which is competitive for real work environments.

### Gain and Inverse Attack

It is well known that SS watermarking is strong against gain attack, however, in order to corroborate that claim an experiment was carried out. After several pirate audio clips were uniformly scaled in the range [0.5,1.5] in 0.1 steps, the detector performance was the same for all of the scaled values.

Due to the linearity of the embedding domain, when the sign of the pirate audio signal is inverted, the same happens in the embedding domain. Therefore, in order to guarantee the correct detection in an inverse attack scenario, the ID in a block is counted when correlation value is bigger that thresholds 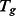 and 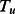 or lower that 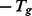 and 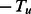, which is a very small change to the detector. This claim was corroborated with several experiments where, in the presence of the inverse attack, the detector performance is not altered

### Comparison

To the best of our knowledge, the work reported in [24] is the only one addressing the collusion-resistant fingerprinting problem with audio signals. Table 3 shows a detailed feature comparison of the proposed system against that proposed in [24]. As it was described in the Related Work section, the work reported in [24] is not robust against sign inversion attack whereas, according to the results described in the Results and Discussion section, the proposed system is robust against this type of attack. A very common real-world audio processing operation is volume gain, the proposed system is able to resist this processing while the work reported in [24] does not. Moreover, unlike [24], this paper reports results for lossy compression and system viability, which are real-world scenarios as music distribution is nowadays in compressed format and real-time. Comparing the work reported in [24] with the proposed system in terms of detection probability per number of colluders is a difficult task as it is unclear which value of false alarm, 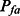, is considered in that work. The aforementioned work lacks in a statistical analysis of the system performance, therefore, a deepest comparison with the proposed system can be biased.

**Table 3:** Comparison between the proposed work and [24].

Algorithm	Robustness			Number of users	System viability
	Lossy compression	Inverse sign	Volume gain		
Tirkel *et al.* [24]	no	no	no	not reported	not reported
Proposed	high quality	yes	yes	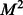	yes

### Summary of Results

In this paper, a block-based approach for fingerprinting is considered. This consideration is due to two facts: 1) a frequency transform for a full typical audio clip is practically intractable and 2) if the fingerprint is replied each block, then, for detection is not necessary the full pirate audio signal. As a consequence of the block-based approach, the detection is carried out according to the half-normal distribution. Through experimentation, it was shown that about 1 second of CD-quality pirate audio signal is enough for probability of colluders detection equal to 1.

The optimal energy for user and group ID fingerprints in function of ODG metric is also studied. It was observed that the bigger user ID fingerprint energy, 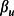, the better detection performance. This characteristic is interesting because users in a group are more likely to collude with each other [17], therefore; the number of group IDs involved in a pirate copy must be minor to the number of colluder IDs.

The impact in the fingerprint detection process of the block length was investigated through experimentation. It was observed that the higher block length the higher collusion-attack robustness. However, for a block length bigger than 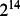 samples the performance improvement is not significative. Moreover, for a bigger block length the needed computing resources are also bigger and even intractable for some platforms.

For validation purposes, the proposed fingerprinting system was implemented in an standard modern computer using free libraries. The performance is guaranteed to be several times better that the real-time restriction. The proposed system viability is demonstrated.

Finally, the robustness of the proposed system to typical attacks in real-world scenarios, such as lossy compression, gain and inverse attacks, was shown. Then results suggest that the proposed fingerprinting system is suitable for practical applications, therefore, attractive for the music industry.

## Materials and Methods

Due to the proposed fingerprinting system utilizes the DCT basis as fingerprint modulators and the insertion domain is the set of MCLT magnitudes, in this section are recalled two Fast Fourier Transform (FFT)-based fast algorithms for MCLT and DCT calculations which are utilized for the proposed fingerprinting system implementation.

### Modulated Complex Lapped Transform

The Modulated Complex Lapped Transform (MCLT) is a particular kind of a 2x oversampled generalized DFT filter bank proposed in [38] whose basis are:
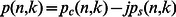
(14)

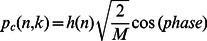
(15)

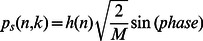
(16)


with:
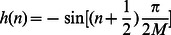
(17)


and
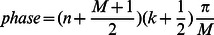
(18)


where 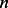 is the time-domain index, 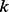 is the frequency-domain index, 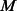 is the sample block length and 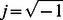. The MCLT coefficients of the input vector 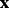 are calculated as 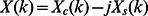 with:
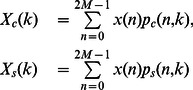
(19)


#### Fast MCLT Algorithm

In [39] it was proposed a FFT-based fast MCLT algorithm. The MCLT coefficients 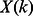 can be obtained as follows:
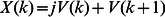
(20)


where
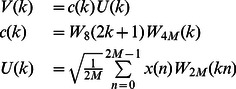
(21)


and 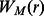 is the common notation for the complex exponential used in Fourier transforms, namely:
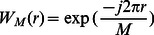
(22)



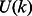
 is a 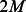 point FFT with orthonormal basis function of the input block 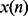, which means that MCLT coefficients can be computed by computing FFT of 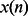 to obtain 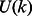 and carring out the operations with factors 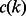.

#### Fast Inverse MCLT Algorithm

In order to carry out the inverse MCLT, in [39] is developed the next relation:
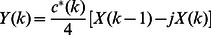
(23)where 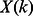 are the MCLT coefficients, the superscript * denotes complex conjugation, and the modulation 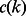 is the same as that in (21). Using (23) we compute the 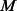 first FFT coefficients of 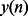, but it is well known that FFT coefficients must satisfy the conjugate symmetry property:
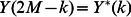
(24)


Finally, we know that 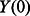 and 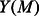 must be real-valued, and after some manipulations:
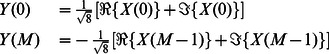
(25)


with 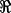 and 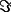 taking the real and imaginary parts, respectively.

### Discrete Cosine Transform

The Discrete Cosine Transform (DCT) is a linear and invertible function in the Real Numbers set, originally derived from Chebyshev polynomials [40]. The DCT basis are orthogonal and defined as follows:
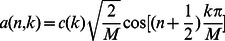
(26)


where
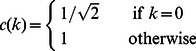
(27)


#### Fast DCT and Inverse-DCT Algorithms

It is known that the Fourier transform of a real-even function 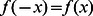 is real-even, and 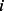 times the Fourier transform of a real-odd function 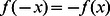 is real-odd, thus for these symmetry conditions it is not necessary to use complex inputs/output. Therefore, it is possible to compute the DCT or the Discrete Sine Transform (DST) by utilizing an FFT algorithm.

Let be the input vector 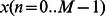 even around 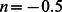 and even around 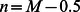, it is possible to show that DFT(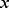) is the non-normalized DCT of 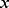, 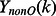 described as follows:
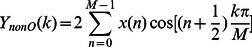
(28)


with basis:
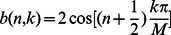
(29)


The basis set described in equation (29) is non-orthogonal, therefore, it is necessary to normalize equation (28) in order to get the orthogonal transform as follows:
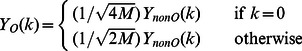
(30)


On the other hand, let be the input vector 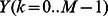 even around 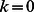 and odd around 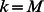, it is possible to show that DFT(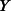) is the non-normalized Inverse DCT of 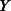, 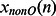 described as follows:
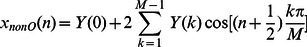
(31)


As in equation (28), it is necessary a normalization procedure in order to get the orthogonal transform. The normalization is carried out as follows:
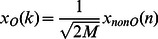
(32)


In the literature, fast algorithms for the DFT have been extensively reported and very efficient software libraries exist [41]. In this work, these libraries are utilized as a module of the DCT and MCLT computing, reducing the effort required for efficient implementation to a butterfly stage implementation for MCLT and a normalization stage implementation for DCT.

### The Fingerprinting System

The frequency domain for embedding is the Modulated Lapped Complex Transform (MCLT). In order to bring perceptual transparency, the fingerprint is embedded into MCLT magnitudes while keeping phases without changes.

#### Fingerprint Embedding

**Figure 11:**
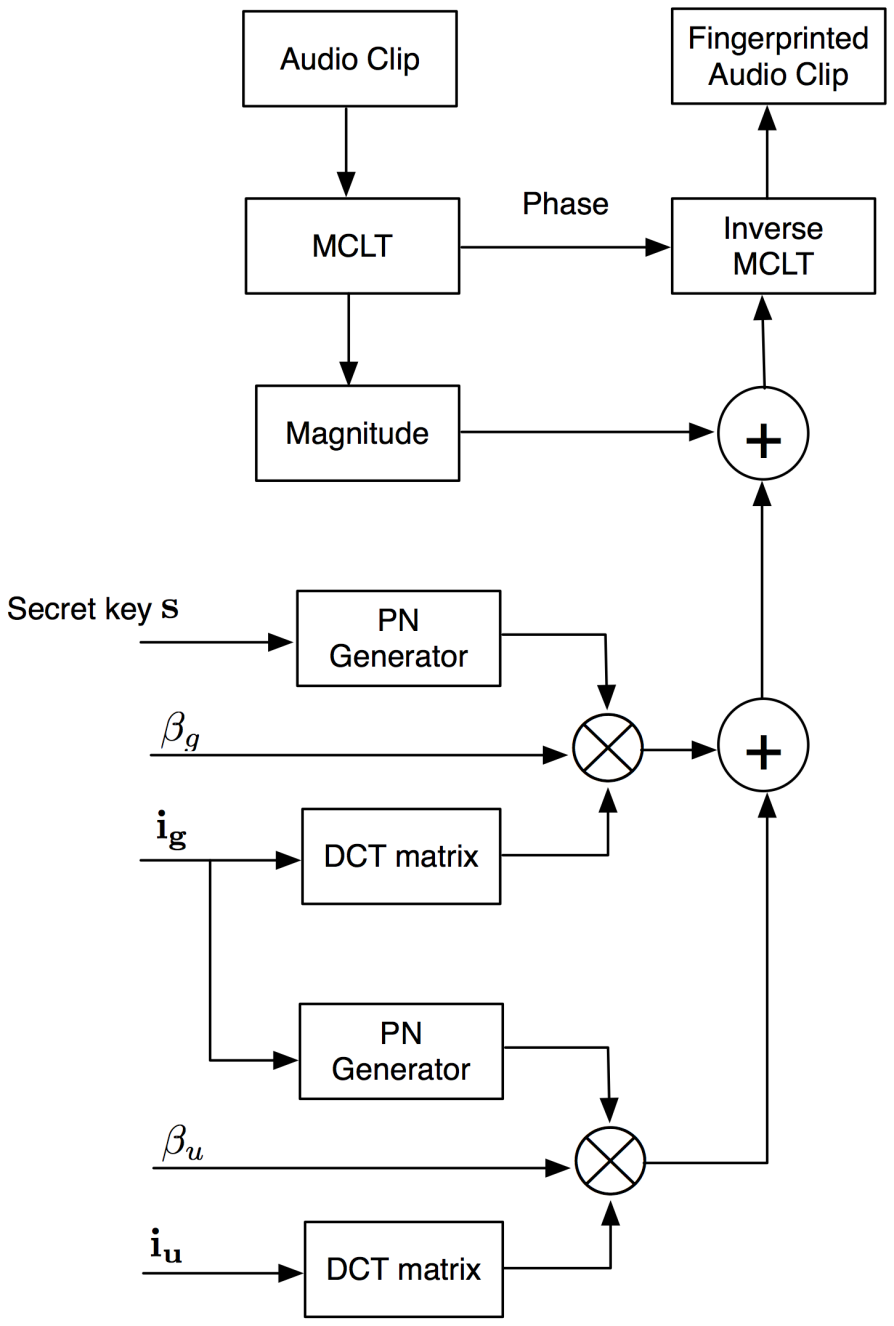
Fingerprint embedding system.

Instead of [18], [19], in this paper the fingerprint is replicated several times along the audio signal in a block-processing fashion as typical CD-quality music clips are conformed by about 8 million of samples and the embedding/detecting process can become intractable if an orthogonal transform is applied to the whole audio clip. Moreover, by splitting the audio signals in blocks for fingerprinting it is possible to detect colluders with a fraction of the whole audio clip which is demonstrated in the Results section. Each samples-block is 50% overlapped as the MCLT is a lapped transform. Due to MCLT is a 2x oversampled DFT filter bank, 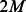 audio samples are required in order to compute 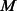 MCLT coefficients. Figure 11 shows a block diagram of the embedding system.

The fingerprint embedding process is carried out as follows: firstly host audio signal is divided into frames of 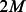 samples per frame. Next, each frame is transformed using the MCLT. Subsequently both magnitude and phase of MCLT are computed. The fingerprint is then added to the MCLT magnitudes while keeping phase without change. The additive technique is utilized for embedding as follows:
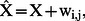
(33)


where 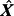 is the fingerprinted MCLT magnitude, 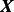 is the original MCLT magnitude and 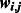 is the fingerprint assigned to the *j*th user of the *i*th group. Finally, inverse MCLT is applied to both processed magnitude and original phase to get the audio signal with hidden fingerprint. The fingerprint is conformed according to equation (8), the secret key 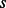 provides the system security in a symmetric-key fashion. The 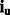 and 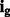 variables represent the authorized user and its group respectively. The PN-Generators produce pseudo-noise with an uniform distribution.

#### Fingerprint Detection

**Figure 12:**
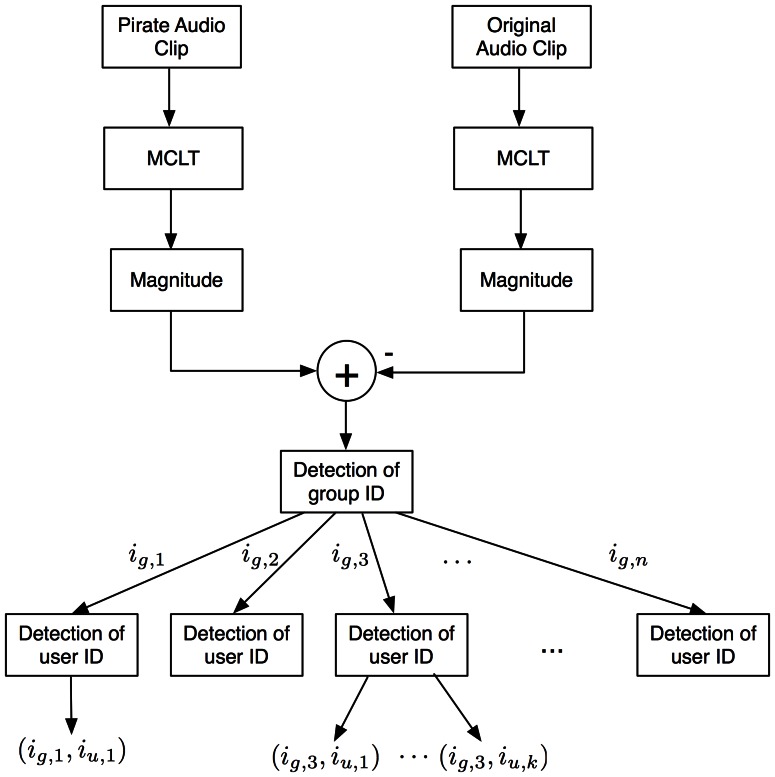
Colluder detection system.

Figure 12 shows the colluders detection system. In fingerprinting systems is a common assumption to get access to the original media. That consideration is taken into account for the proposed system.

Detection procedure is carried out in a block fashion as the fingerprint is embedded in the same way. In this paper, a detection strategy using several MCLT magnitude blocks is proposed.

#### Group ID Detection

**Figure 13:**
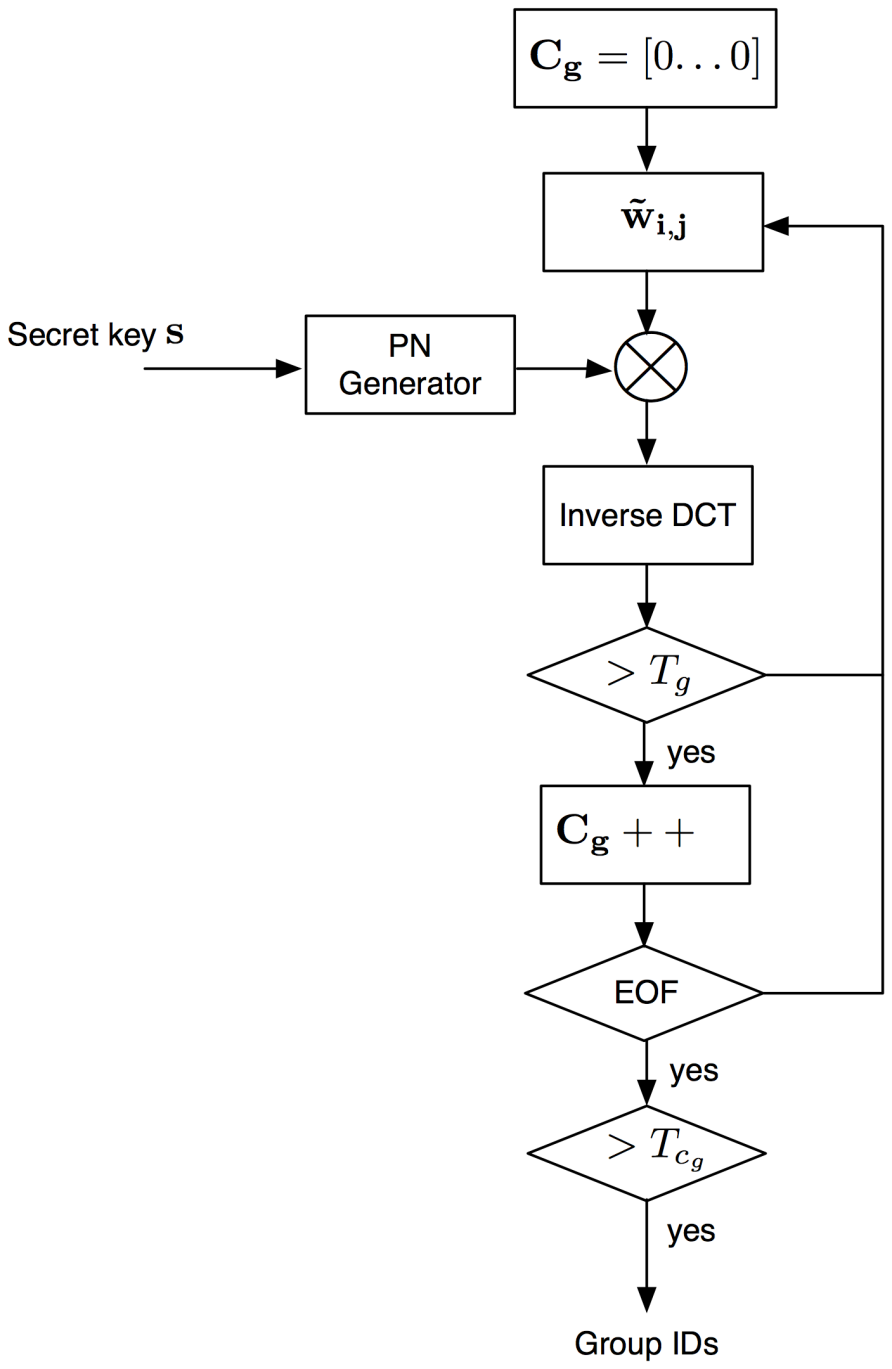
Group IDs detection system.

Figure 13 shows the group IDs detection system. For each available MCLT coefficients block, group detection is carried out according to the threshold, 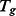, described in equation (12). For the whole pirate audio clip, there is a counter vector 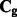 that registers the number of times that each component of 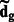 exceeds 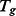.

**Figure 14:**
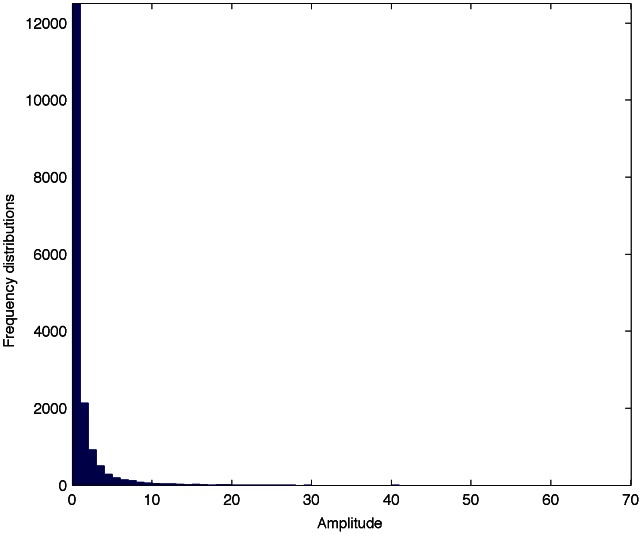
Distribution of the counter vector 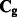.

Instead of the group ID detection in each block where the threshold is computed assuming a Gaussian distribution, the threshold, 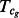, for detection in the counter vector 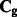 must consider other distribution as the lower limit of that distribution will always be zero. In order to know the statistical behavior of the counter vector 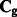, 120 different fingerprinted audio clips are utilized. Figure 14 shows the distribution of the counter vector 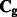.

As can be seen from Figure 14, 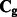 can be modeled by the Half-normal distribution, which is defined as follows:
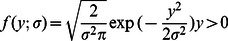
(34)


with cumulative distribution function 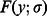 as follows,
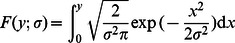
(35)


For a given threshold, 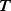, the false detection probability 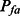, is computed by subtracting the cumulative distribution function to the unit as follows:
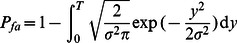
(36)


**Figure 15:**
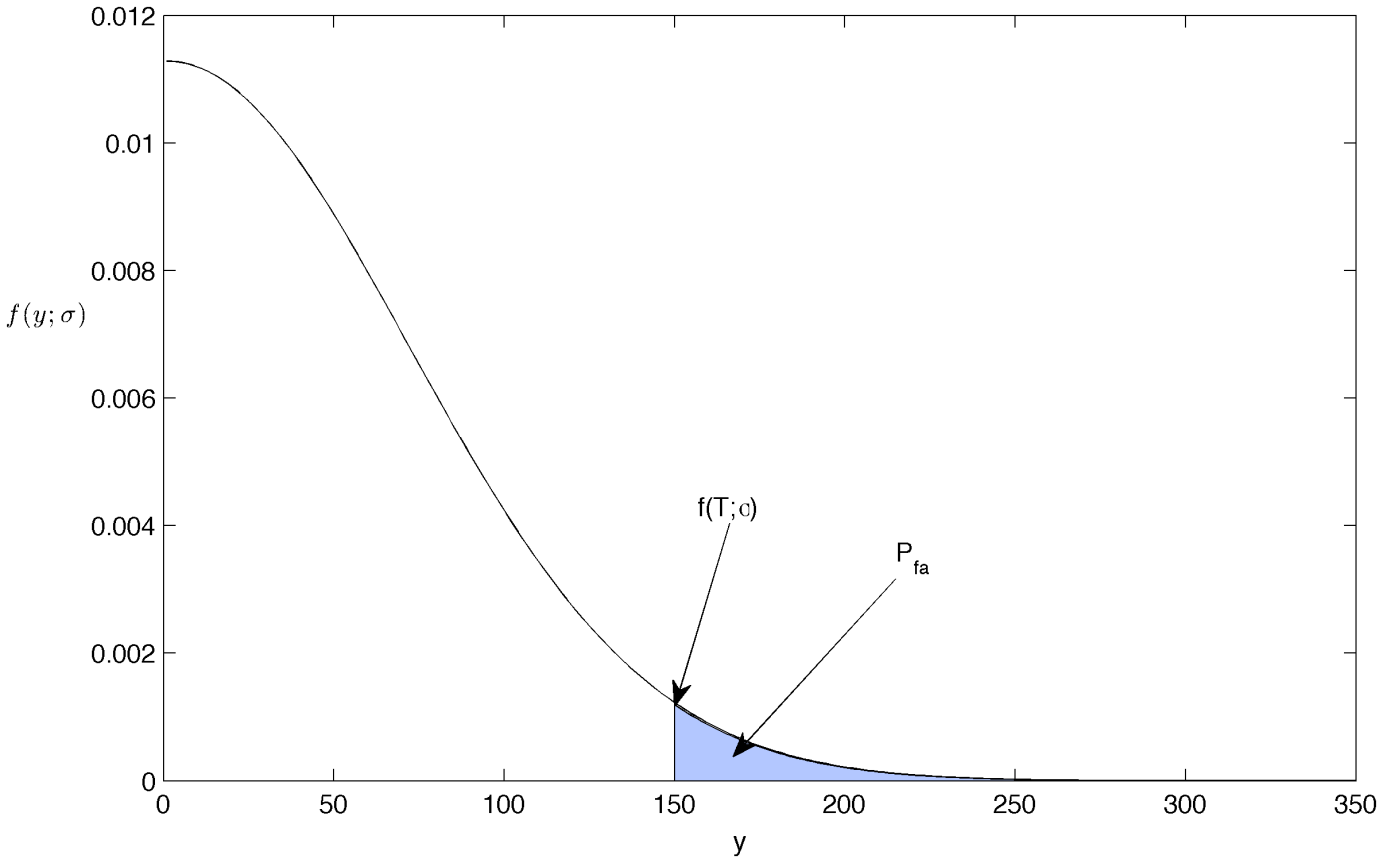
Half-normal distribution.

Figure 15 shows the 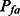 for a given threshold 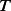 in a Half-normal distribution.

Using a change of variable 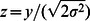 in equation (35) it becomes:
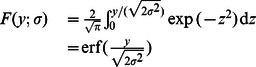
(37)


where erf(.) is the error function and is related to the complementary error function as:
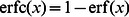
(38)


From equations (36), (37) and (38); the false detection probability 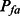 for a given threshold 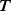 can be rewritten as:
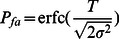
(39)


Therefore, the threshold, 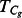 for a 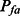 given for a group ID detection in a pirate audio clip can be computed as follows:
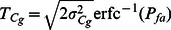
(40)


where 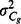 is the variance of 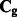.

#### User ID Detection

**Figure 16:**
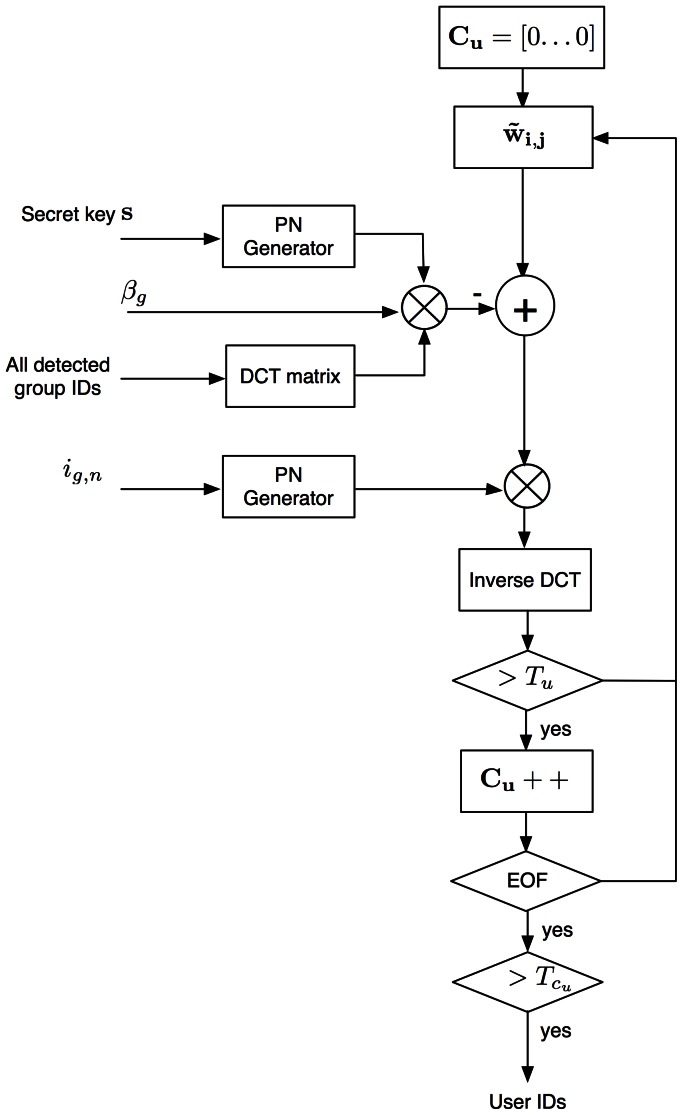
User IDs detection system.



Figure 16 shows the user ID detection system. In similar form that group ID detection, for each available MCLT coefficients block, user detection is carried out according to the threshold, 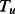, described in equation (13). For the whole pirate audio clip, there is a counter vector 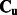 that registers the number of times that each component of 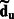 exceeds 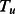. Counter vector 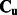 is modeled as a Half-normal distribution and the corresponding threshold 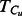 for a given 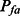 is calculated according to:

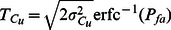
(41)where 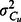 is the variance of 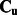. In order to improve user ID detection, the interference due to group ID is previously removed.

#### Number of Users

Due to fingerprints are formed by two DCT modulated PN-sequences, the number of possible IDs for each of them is equal to their respective lengths, 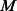 for both. Therefore, the maximum number of possible users of the system is 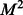.

### Software Implementation

Audio signals are processed as vectors of float numbers in the range 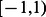. For audio file manipulations the libsndfile library [42] is used. The entire fingerprinting system was implemented in C language in a Intel Core i7 CPU and 8 GB RAM. The compiler used in this work is GCC version 4.2.1 and the operating system is Mac Os X version 10.7.5. The programs are compiled with -o3 optimization flag. In order to compute the FFT for the fast MCLT and DCT algorithms described above, the FFTW library [41] is utilized.

FFTW is an optimized library that implements most of the variants of the Discrete Fourier Transform. Moreover, FFTW is able to exploit the Message Passing Interface (MPI) and multi-threaded strategies in order to utilize the full power of modern personal computers. Due to the computer used for validation of the proposed fingerprinting system is a multi-core shared-memory computer, the instantiation of FFTW is carried out using multi-threaded calls.

## Conclusions

In this paper, a collusion-resistant fingerprinting system for audio signals is proposed. Each fingerprint is formed by a PN-sequence representing a group ID and other representing one user ID, following state-of-the-art ideas for fingerprinting systems in digital images. Due to nature of audio signals, the fingerprint is replied several times along the audio clip, therefore, it is not necessary the whole audio clip in the detection process. This characteristic guarantees the performance to be several times better that the real-time restriction. The detector performance after high quality lossy compression remains competitive for real work environments. The number of users available, the low computational complexity and the high quality lossy compression robustness make the proposed algorithm attractive for a number of audio processing applications.
